# Does real-time objective feedback and competition improve performance and quality in manikin CPR training – a prospective observational study from several European EMS

**DOI:** 10.1186/s13049-015-0160-9

**Published:** 2015-10-15

**Authors:** JR Smart, K. Kranz, F. Carmona, TW Lindner, A. Newton

**Affiliations:** Research Consultant for South East Coast Ambulance NHS Trust (SECAmb), Banstead, UK; Swiss Institute of Emergency Medicine (SIRMED), Nottwil, Switzerland; Sistema Emergencias Mediques (SEM), Barcelona, Spain; Norwegian Air Ambulance Foundation, Drøbak, Norway; SAFER (Stavanger Acute medicine Foundation for Education and Research) and Stavanger University Hospital, Stavanger, Norway; South East Coast Ambulance NHS Trust (SECAmb), Banstead, UK

**Keywords:** Manikin CPR training, Adult and infant, Real-time objective feedback, Competition

## Abstract

**Background:**

Previous studies have reported that the quality of cardiopulmonary resuscitation (CPR) is important for patient survival. Real time objective feedback during manikin training has been shown to improve CPR performance. Objective measurement could facilitate competition and help motivate participants to improve their CPR performance. The aims of this study were to investigate whether real time objective feedback on manikins helps improve CPR performance and whether competition between separate European Emergency Medical Services (EMS) and between participants at each EMS helps motivation to train.

**Methods:**

Ten European EMS took part in the study and was carried out in two stages. At Stage 1, each EMS provided 20 pre-hospital professionals. A questionnaire was completed and standardised assessment scenarios were performed for adult and infant out of hospital cardiac arrest (OHCA). CPR performance was objectively measured and recorded but no feedback given. Between Stage 1 and 2, each EMS was given access to manikins for 6 months and instructed on how to use with objective real-time CPR feedback available. Stage 2 was undertaken and was a repeat of Stage 1 with a questionnaire with additional questions relating to usefulness of feedback and the competition nature of the study (using a 10 point Likert score). The EMS that improved the most from Stage 1 to Stage 2 was declared the winner. An independent samples Student t-test was used to analyse the objective CPR metrics with the significance level taken as *p* < 0.05.

**Results:**

Overall mean Improvement of CPR performance from Stage 1 to Stage 2 was significant. The improvement was greater for the infant assessment. The participants thought the real-time feedback very useful (mean score of 8.5) and very easy to use (mean score of 8.2). Competition between EMS organisations recorded a mean score of 5.8 and competition between participants recorded a mean score of 6.0.

**Conclusions:**

The results suggest that the use of real time objective feedback can significantly help improve CPR performance. Competition, especially between participants, appeared to encourage staff to practice and this study suggests that competition might have a useful role to help motivate staff to perform CPR training.

## Background

Previous studies have reported that the quality of cardiopulmonary resuscitation (CPR) is an important component of patient survival from out-of-hospital cardiac arrest (OHCA) [1]. There is evidence that scenario training and objective CPR feedback improves CPR quality amongst healthcare providers [2]. In Europe, there are approximately 350,000 deaths each year following unsuccessful CPR [3]. In 2003 the ILCOR Advisory Statement outlined a number of factors which were important for patient survival [4]. The Utstein Formula for Survival was derived from this work during a symposium in May 2006 [5]. The participants of the symposium stated that the greatest need for improvement of survival was educational efficiency and local implementation. Recent statements suggest that an improvement in resuscitation quality continues to be dependent on improving effective implementation and engendering a culture of quality improvement [6].

In 2012 a group of European pre-hospital professionals and several Emergency Medical Service (EMS) organisations supported by the Society in Europe for Simulation Applied to Medicine, formed a network, the Pre-Hospital Special Interest Group (PH-SIG). The aim of the group is to share experience in the use of simulation for pre-hospital education and quality improvement [7]. The use of simulated OHCA training scenarios is one aspect of best practice sharing that PH-SIG can help facilitate.

In recent years, training to improve quality in CPR has benefited from the introduction of devices and manikins, which provide real time measurement of key metrics in CPR [8, 9]. The key metrics of CPR performance are chest compression depth and rate, full release between compressions (leaning), no-flow time and ventilation volume and rate. These parameters can now be accurately and objectively measured in real time and used as immediate feedback for the trainee to help improve performance.

The hypothesis of the study was:

- Real time objective feedback whilst manikin training for EMS teams improves CPR performance

Historically, competition between organisations and individuals has helped to improve performance and its use has been shown to be beneficial in healthcare [10]. The European Resuscitation Council (ERC) has organised an Advanced Life Support (ALS) competition for teams representing different healthcare organisations during their annual scientific congress annually since 2013 [11].

The primary objective of the study was:

- To analyse whether real time objective feedback during CPR training can improve CPR quality amongst professional pre-hospital EMS healthcare providers.

The secondary objectives of the study are:

- To investigate if competition between the participating EMS sites had an effect on their motivation to train and improve performance.
- To investigate if competition between the participating professional pre-hospital EMS healthcare providers at each site had an effect on their motivation to train and improve performance.

The study objectives were intended to help the participating EMS sites improve two of the factors from the Utstein Formula for Survival: educational efficiency (by means of objective feedback) and local implementation (by introducing competition).

## Methods

The study was carried out in two Stages. Stage 1 was to objectively assess the CPR performance of EMS professionals from 10 different study sites in Europe using a standardised protocol and identical manikins to simulate both an adult and infant (6 month, 5–6 Kg) OHCA scenario. Stage 2 was to re-assess the CPR performance using the same standardised protocol six months later, after real time CPR feedback training had been made available. The competitive nature of the study was stated at the outset to all participants and the site demonstrating the greatest improvement in their overall CPR performance for adult and infant assessments at Stage 2 compared with Stage 1 would be declared the winner. The scoring for the competition would be the ‘Overall CPR score’ calculated and provided by the equipment after each assessment. This score is calculated using an algorithm developed in close collaboration with members of the AHA ECC Sub-committee and co-authors of the 2013 AHA Consensus Statement [1, 12]. It uses a combined weighted score for each of the measured key CPR metrics performed on the manikin to produce the ‘Overall CPR score’. The parameters differ for the adult and paediatric manikin assessments according to the relevant ERC 2010 Resuscitation Guidelines. The key CPR metrics used in the algorithm relate to compression quality (depth, rate, full release, hand position, no flow time, compression number per cycle) and ventilation quality (volume and rate). If CPR was carried out during the assessments fully in accordance with the Resuscitation Guidelines, then 100 % was scored. If the CPR deviated from the Resuscitation Guidelines, then the score was reduced. The larger the deviation, the more the score is reduced. PH-SIG considered this ‘Overall CPR score’ to be an objective and consistent determinant of overall CPR performance for the competition between the 10 sites.

Stage 1 of the study took part between April and July 2014. All 10 EMS sites were visited during this time and a local site co-ordinator invited 10 crews (two persons per crew) to be briefed and ready for the Stage 1 assessments. Each participant was asked to complete a short 10 question anonymised questionnaire (translated to the language used at the sites). This recorded participant demographic (age, sex, height) and professional experience information (job title, experience in job, time since professional education, duration of professional education, time since CPR assessment and OHCA response in the last 12 months). The adult OHCA scenario protocol included a timed 30m transit on foot for each crew to simulate leaving their ambulance to the patient. Upon arrival, each of the two-crew members performed chest compressions or ventilations using a standardised Bag Valve Mask (BVM) according to ERC (2010) Adult BLS Guidelines (30 compressions and 2 ventilations) for 2 min until asked to stop [13]. The crew members were then asked to swap tasks and repeat the full protocol (including the 30 m transit). No feedback about their CPR performance was given during or after the assessment. The objective results were recorded for later analysis.

The same crews then repeated the assessment using the infant manikin, performing an infant OHCA scenario according to ERC (2010) Paediatric/Infant BLS Guidelines (15 compressions and 2 ventilations) using a standardised paediatric Bag Valve Mask (BVM) but without the five initial rescue breaths, for 2 min [14]. The same protocol as the adult OCHA scenario was followed and the 30 m transit was carried out together with the swapping of tasks and recording of results.

Each EMS site was left with identical equipment as used in the Stage 1 assessments and each site co-ordinator was given an introductory briefing of how to set up and use the equipment. The site co-ordinator was also tasked with ‘encouraging’ and motivating their crews to practice with the equipment using real time objective feedback switched on before Stage 2 of the Study. A practice was defined as use of a manikin with objective feedback switched on for at least 2 min duration and to include chest compressions and ventilations. In order to focus their training, the site co-ordinators were also given a summarised report of their specific sites objective CPR metrics performance for Stage 1 together with the overall mean values for all the 10 sites.

Stage 2 took place between October 2014 and January 2015. The 10 sites each had 6 months to use the equipment (with feedback on) after Stage 1 was completed. Stage 2 was a repeat of Stage 1. The participants in Stage 2 must have had the opportunity to practice on the manikins left in the intervening period after Stage 1. Participants completed an extended anonymised questionnaire which repeated the same questions from Stage 1. This questionnaire also asked the participants additional questions with regard to how many times they had used the manikins between Stage 1 and Stage 2 and their perceptions of the value of objective feedback during training together with the influence that competition had on their motivation to train. Participants were asked to score each of these additional questions using a 10 point Likert score [15]. A standardised 19 question discussion guide was also used to obtain additional qualitative feedback from each of the site co-ordinators and to supplement the Stage 2 questionnaire data.

Following Stage 2, the site co-ordinators were given the summarised results for all sites and the identity of the winner of the ‘competition’. The winner was rewarded with keeping the equipment for their site.

### Participants

The PH-SIG helped identify the European EMS sites to take part in this study (see Table 1). The sites are different in terms of size, structure and Front Line staff (FL) composition and geographic coverage. PH-SIG requested that pre-hospital professional participants had to be ‘active’ members of their designated EMS at the time of the study and attend OHCAs as part of their duties. The site co-ordinators were responsible for recruiting the participants in Stage 1 and for Stage 2. PH-SIG requested that pre-hospital professional participants had to be ‘active’ members of their designated EMS at the time of the study and attend OHCA’s as part of their duties. Informed consent was obtained for all the participants taking part in Stage 1 and Stage 2 of the study.

**Table 1:** Key EMS site information (see Acknowledgements for reference source)

	Population	KM2	Predominantly	Resus. calls/year	Number FL Staff	FL Staff comprising
BSPP - Paris - France	6,650,000	762	Urban	3126	8117	62 Doctors, 55 Nurses, 8000 Technicians (Firefighters)
Filipstad - Sweden	16,000	2000	Rural	9	21	8 Advanced Paramedics, 10 Nurses, 3 Technicians
SECAmb HART - Gatwick - UK	4,600,000	5700	Mixed	2627	2056	150 Advanced Paramedics, 1000 Paramedics, 906 Technicians
SEM - Barcelona - Spain	1,600,000	102	Urban	431	144	33 Doctors, 51 Nurses, 60 Technicians
Rettung - Chur - Switzerland	86,000	875	Rural	50	28	2 Doctors, 3 Advanced Paramedics, 20 Paramedics, 3 Technicians
SALVA - Locarno - Switzerland	150,000	1120	Rural	66	40	1 Doctor, 5 Advanced Paramedics, 31 Paramedics, 3 Technicians
RAVU - Amersfoort - Holland	1,200,000	1385	Mixed	500	275	150 Nurses, 125 Technicians
EMS - Copenhagen - Denmark	1,700,000	2568	Mixed	1400	450	60 Doctors, 150 Paramedics, 240 Technicians
INEM - Lisbon City - Portugal	600,000	85	Urban	1059	245	33 Doctors, 33 Nurses, 179 Technicians
DRK - Hofgeismar - Germany	60,000	800	Rural	72	54	2 Doctors, 1 Advanced Paramedic, 36 Paramedics, 15 Technicians

### Equipment

The equipment used for the study was the adult Laerdal Resusci Anne QCPR® torso manikin, the infant Resusci Baby QCPR® manikin and the SimPad® SkillReporter.

The adult manikin was fitted with a chest spring requiring 60Kg of elastic force (Kgf) to compress the chest 5 cm for both Stage 1 and 2. This was representative of a stiffer than average adult chest but still within the normal range of chest stiffness reported [16].

In addition to the equipment listed, a standardised adult Bag Valve Mask (BVM) was used in the study for the adult CPR assessment and a standardised paediatric BVM for the infant assessments. The same equipment was used to record the assessments for all 10 participating EMS sites for both Stage 1 and Stage 2 of the Study.

### Statistical analysis

An independent samples Student’s t-test and Chi square Asympt. Sig 2-sided test were used to analyse the questionnaire information. The mean objective CPR scores and the 95 % Confidence Intervals (CI) calculated for both Stages were analysed using an Independent samples Student’s t-test. The significance level was taken as *p* < 0.05. Statistical analysis was done through SPSS 19.0 software (IBM SPSS Statistics for Windows, Version 19.0. Armonk, NY: IBM Corp.).

The sample size of 10 EMS sites and 10 teams per site for Stage 1 and Stage 2 was for convenience as there were resource limitations and practical constraints with the running of this study. The control group for this study were those taking part in Stage 1 and the intervention group were those taking part in Stage 2.

## Results

### Demographics

Analysis of the questionnaire data presented as Table 2 suggests that there was no statistically significant difference between the ages, heights, sex and overall professional experience and training of the participants in both stages at each site. Questionnaire data from Stage 2 suggested that 155 participants took part in both Stage 1 and Stage 2 and 45 participants took part in Stage 2 only. These 45 participants were relatively uniformly spread amongst the 10 centres. The data also suggested that more Doctors and Nurses took part in Stage 1 than Stage 2 and more Paramedics took part in Stage 2 than Stage 1.

**Table 2:** Questionnaire demographic data

	Stage 1	Stage 2	*p* value
Age (yr) mean	37.5	36.35	0.232
Age (yr) SD	9.6	9.40	
Height (cm) mean	176.85	175.55	0.188
Height (cm) SD	8.46	7.98	
Males (n)	153	141	0.174
Females (n)	47	59	
Experience in job			0.685
Time since professional education			0.558
Duration of professional education			0.472
Time since last CPR assessment			0.528
Attend at least 1 OHCA in last 12 months			0.587

### Adult assessment

**Fig. 1:**
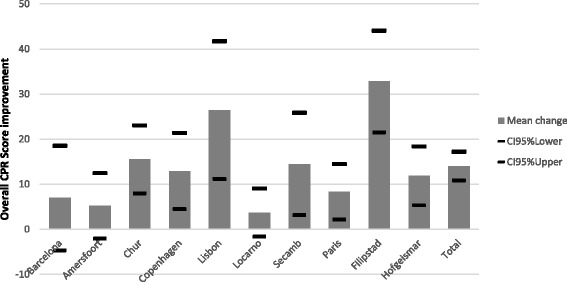
Adult assessment – mean overall CPR score improvement per site from Stage 1 to Stage 2, with upper and lower confidence limits (95 %)

The mean improvement of the overall CPR score per site for the adult assessment with upper and lower confidence limits (95 %) is presented as Fig. 1. The specific CPR metrics measured for the adult assessment are presented in Table 3. The variability in the mean Overall CPR score for the adult assessment achieved by each of the 10 sites at Stage 1 and Stage 2 is presented as Fig. 3.

**Table 3:** Adult assessment — CPR metrics for Stage 1 and Stage 2

		Adult (*n* = 200) Stage 1		Adult (*n* = 200) Stage 2					
		Mean	SD	Mean	SD	Mean diff.	CI 95 % low	CI 95 % up	*p* value
Overall CPR score	%	81.0	20.9	95.1	8.9	14.0	10.9	17.2	0.00
Compression depth average	mm	51.4	7.9	58.8	5.3	7.4	6.1	8.8	0.00
Compression rate average	cpm	116.4	13.8	111.8	7.3	−4.6	−6.7	−2.4	0.00
Leaning average	mm	3.3	1.9	2.4	1.4	−1.0	−1.3	−0.6	0.00
Flow fraction	%	83.4	3.8	84.7	3.1	1.3	0.7	2.0	0.00
Ventilation volume average	ml	438.1	147.1	488.8	130.8	50.7	23.4	78.1	0.00
Ventilation rate average	vpm	5.3	1.7	5.7	1.3	0.4	0.1	0.7	0.00
Average interuption (no flow time)	sec	3.3	0.9	3.1	0.7	−0.3	−0.4	−0.1	0.00

**Table 4:** Infant assessment — CPR metrics for Stage 1 and Stage 2

		Infant (*n* = 200) Stage 1		Infant (*n* = 200) Stage 2					
		Mean	SD	Mean	SD	Mean diff.	CI 95 % low	CI 95 % up	*p* value
Overall score	%	55.1	21.2	86.5	12.1	31.4	28.0	34.8	0.00
Compression depth average	mm	31.9	8.7	42.7	3.1	10.8	9.5	12.1	0.00
Compression rate average	cpm	127.2	20.9	116.7	9.0	−10.5	−13.7	−7.3	0.00
Leaning average	mm	3.7	2.5	1.9	1.3	−1.8	−2.2	−1.4	0.00
Flow fraction	%	71.6	8.5	77.1	4.8	5.4	4.1	6.8	0.00
Ventilation volume average	ml	38.1	11.1	42.3	15.8	4.2	1.5	6.9	0.00
Ventilation rate average	vpm	11.6	2.5	10.7	2.5	−0.9	−1.4	−0.4	0.00
Average interuption (no flow time)	sec	2.9	1.2	2.4	0.7	−0.5	−0.7	−0.3	0.00

Table 3 presents the overall mean data for all 10 EMS sites for the adult assessment. The Table presents the key CPR metrics recorded for both stages of the study. The results suggests that the overall percentage score of CPR performance together with all the listed individual CPR metric measurements significantly improved according to ERC (2010) Adult BLS Guidelines [13] in Stage 2. The overall mean depth of compressions increased by 7.4 mm in Stage 2 and the rate reduced to 112 per minute. Mean overall leaning was reduced (−1.0 mm) in Stage 2 and the mean overall flow fraction improved to 84.7 %. The mean overall ventilation volume also increased significantly by 50.7 ml in Stage 2 and the rate significantly reduced to 5.7 per minute with a smaller variability between participants (sd = 1.7 in Stage 1 compared with sd = 1.3 in Stage 2). The reduction in hands off time (also known as ‘no flow time’) between Stage 1 and Stage 2 was also significant.

### Infant assessment

**Fig. 2:**
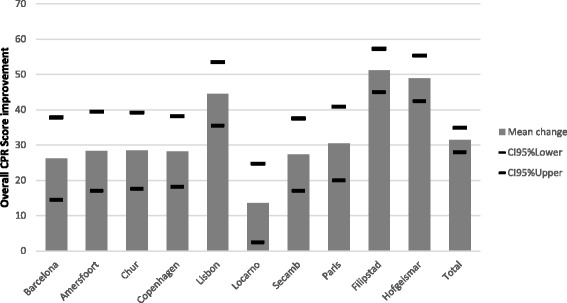
Infant assessment — mean overall CPR score improvement for each site from Stage 1 to Stage 2, with upper and lower confidence limits (95 %)

The mean improvement per site for the infant assessment with upper and lower confidence limits (95 %) is presented as Fig. 2. The specific CPR metrics measured for the infant assessment are presented in Table 4. The variability in the mean Overall CPR score for the infant assessment achieved by each of the 10 sites at Stage 1 and Stage 2 is presented as Fig. 4.

The overall mean data for all 10 EMS sites for the infant assessment is presented as Table 4. The mean overall percentages score of CPR performance and the mean individual objective CPR metric measurements significantly improved according to ERC (2010) Paediatric/Infant Guidelines [14] in Stage 2. The overall mean depth of compressions increased by 10.8 mm in Stage 2 achieving an overall mean depth of 42.7 mm (the ERC (2010) Paediatric/Infant Guidelines state a depth of 1/3 AP which on this manikin is 38 mm). The rate reduced significantly to 117 per minute (from 127). Mean overall leaning was reduced (−1.8 mm) in Stage 2 and the mean overall flow fraction improved to 77.1 %. The mean overall ventilation volume increased significantly to 42.3 ml (sd = 15.8) and the rate reduced significantly to 10.7 per minute (from 11.6 per minute). The reduction in hands off time from Stage 1 to Stage 2 was also significant. The larger standard deviation in ventilation volume recorded at Stage 2 (sd = 15.8 ml), however, suggests that there was greater variability amongst the participants than Stage 1.

The questions from the Stage 2 questionnaire are listed in Table 5 with the mean and standard deviation results from the 10 point Likert scoring. Table 5 suggests that the question relating to the first study aim (usefulness of objective feedback for CPR) was positive overall (mean score of 8.5, sd = 1.8) and the manikins were thought relatively easy to use (mean score of 8.2, sd = 1.7). It appears that the questions relating to the second study aim (motivation because of competition between EMS sites and competition within EMS sites) was less positive with overall mean scores of 5.8 (sd = 2.9) and 6.0 (sd = 2.7) respectively. Table 5 also includes a Yes/No answer obtained from the individual EMS study site co-ordinator using the standardised discussion guide.

**Table 5:** Stage 2 Questionnaire and discussion guide information

	How easy was it to practice on the manikins left after Stage 1? (very diff = 1; very easy = 10) Participant answer (*n* = 200)		Did the competition between countries motivate you to practice? (1 = not at all; 10 = very much) Participant answer (*n* = 200)		Did the competition between countries motivate teams to practice? Site co-ordinator perception (*n* = 1 per site)	Did the competition between colleagues motivate you to practice? (1 = not at all; 10 = very much) Participant answer (*n* = 200)		Did the competition between colleagues motivate teams to practice? Site co-ordinator perception (*n* = 1 per site)	Did the objective feedback help you improve your CPR performance? (1 = not at all; 10 = very much) Participant answer (*n* = 200)		Did the objective feedback help motivate teams to practice? Site co-ordinator perception (*n* = 1 per site)
	Mean	SD	Mean	SD		Mean	SD		Mean	SD	
Barcelona	7.6	1.8	6.4	2.9	No	5.9	2.4	Yes	8.9	2.1	Yes
Secamb	7.9	2.1	4.5	3.0	No	5.4	3.0	Yes	8.9	2.1	Yes
Locarno	9.1	1.8	7.0	1.6	No	7.0	2.1	Yes	8.5	2.5	Yes
Lisbon	8.1	2.3	6.3	3.0	No	5.5	2.9	Yes	8.8	1.8	Yes
Hofgeismar	8.1	2.4	5.1	2.1	Yes	5.5	2.5	Yes	8.3	2.7	Yes
Paris	9.1	1.7	7.1	2.1	Yes	7.4	2.5	Yes	8.7	1.7	Yes
Copenhagen	8.5	2.2	6.9	2.6	Yes	7.2	2.6	Yes	8.7	2.0	Yes
Amersfoort	8.0	1.7	3.7	2.7	No	4.4	2.5	N/A	8.3	1.8	Yes
Filipstad	7.6	2.5	8.0	2.5	Yes	8.0	2.1	Yes	9.0	2.1	Yes
Chur	7.7	2.0	2.8	2.2	No	3.7	2.4	Yes	7.3	2.9	Yes
Overall	8.2	1.7	5.8	2.9		6.0	2.7		8.5	1.8	

The questionnaire for Stage 2 also included questions that asked the participants to estimate how often they thought they had practiced on the adult and the infant manikins in the 6 month period between Stages. Table 6 presents the mean data for each EMS site and also presents the overall mean results for all sites. The overall mean result suggests that the participants appeared to have practiced more frequently with the adult manikin in the 6 month period than with the infant manikin (mean = 6.2 and 5.5 respectively). It can be seen however that there is a wide variability between the EMS sites, for example, Filipstad reporting a mean usage of 15.6 for the adult manikin and 15.8 for the infant manikin and Barcelona reporting a mean usage of 2.3 and 1.8 respectively. This wide variability is reflected in the overall standard deviation of the mean of 6.8 for the adult and 6.2 for the infant manikin. Table 6 also includes the corresponding number for ‘perceived usage’ for each EMS site and for each manikin type. This was provided by the relevant EMS study site co-ordinator using the standardised discussion guide. Their perception was generally relatively accurate when compared to the participant answer although the Locarno site co-ordinator overestimated the participant manikin use and the Chur EMS site co-ordinator underestimated the participant manikin use.

**Table 6:** Number of ‘practices’ and perceived usage data

	Number of adult practices between Stage 1 and Stage 2 Participant answer (*n* = 20 per site)		Number of adult practices between Stage 1 and Stage 2 site co-ordinator perception (*n* = 1 per site)	Number of paediatric practices between Stage 1 and Stage 2 Participant answer (*n* = 20 per site)		Number of paediatric practices between Stage 1 and Stage 2 site co-ordinator perception (*n* = 1 per site)
	Mean	SD		Mean	SD	
Barcelona	2.3	1.6	3	1.8	1.0	3
Secamb	4.6	5.8	5	3.9	5.2	5
Locarno	8.4	8.2	10	3.6	2.6	10
Lisbon	5.6	3.5	NA	5.5	3.9	NA
Hofgeismar	10.6	7.3	10	9.9	6.9	10
Paris	4.8	4.0	5	4.3	2.7	5
Copenhagen	2.8	2.7	3	2.5	2.6	3
Amersfoort	1.9	1.3	2	2.0	2.0	2
Filipstad	15.6	9.8	15	15.8	9.9	15
Chur	6.1	4.4	3	6.0	4.2	3
Overall	6.2	6.8		5.5	6.2	

**Fig. 3:**
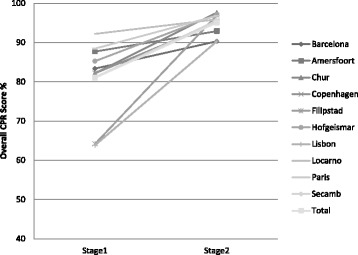
Adult assessment — mean overall CPR score for each site for Stage 1 and Stage 2

**Fig. 4:**
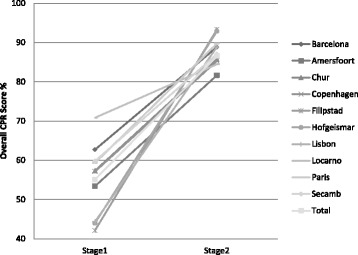
Infant assessment — mean overall CPR score for each site for Stage 1 and Stage 2

Figures 1 and 2 suggest that the Filipstad site improved the most (mean overall CPR score) from Stage 1 to Stage 2 for both the adult and the infant assessments and therefore were declared the ‘winners’ of the competition. The team at Filipstad also appeared to have practiced the most on both manikins. The mean overall CPR scores for each site for Stage 1 and Stage 2 are also presented in graphical form in Fig. 3 (adult assessment) and Fig. 4 (infant assessment).

## Discussion

### Objective feedback

The most important finding from this study suggests that objective real time feedback whilst undertaking CPR manikin training appears to significantly improve adult and infant CPR performance.

The questionnaire demographic data suggests that there was no significant difference between participants taking part in Stage 1 and 2. This is important, as previously published studies have suggested that these factors can make a difference in CPR performance [17–20].

The mean results presented for the adult manikin assessment suggest that there was a significant improvement of all the measured CPR metrics. The mean overall CPR score improvement for all sites for the adult assessment was also significant. However, the improvement was not significant at Barcelona, Amersfoort and Locarno. Information from the site co-ordinators at Barcelona and Amersfoort suggest that the participants at these sites practiced on the manikins the least between Stage 1 and Stage 2 and at Locarno the Stage 1 mean overall score was the highest recorded (92 %). This initial high score improved to 96 % at Stage 2 but this was not significant. Fig. 3 illustrates the mean overall CPR score for each site for Stage 1 and Stage 2 and it is clear that the Locarno site had a lower potential to improve in Stage 2 even though the EMS crews at Locarno practised relatively frequently (over 8 times). The largest improvements were seen at Filipstad and Lisbon. At Filipstad the site co-ordinator reported that each participant had practiced the most (over 15 times) between Stage 1 and 2 (compared to an average of 6.2 for all sites). At Lisbon the Stage 1 mean overall score was initially relatively low (64 %) improving to 90 % at Stage 2. The mean result from Stage 2 for all sites for the adult manikin assessment suggests that good quality chest compressions were being performed with reduced variability in chest compression depth [21]. This was also the case with the ventilations with reduced variability in volume and rate delivered.

The mean results presented for the infant manikin assessment suggest that there was also a significant improvement for all the individual measured CPR metrics. The mean ventilation volume increased to 42 ml at Stage 2 but with a greater standard deviation (sd = 15.8) suggesting that there was increased variability between participants. The mean overall CPR score improvement for all sites for the infant assessment was significant and this was true for all the individual EMS sites as well. The greatest improvement was seen at Filipstad, Hofgeismar and Lisbon. At Filipstad and Hofgeismar the site co-ordinators reported that the participants had practiced 15.8 and 10.6 times respectively between Stage 1 and Stage 2 (compared to an average of 6.2 for all sites). At Lisbon the Stage 1 mean overall score was initially relatively low (44 %) improving to 89 % at Stage 2 but the team there do not appear to have practiced as frequently on the infant manikin as on the adult manikin. The mean results from Stage 2 for all sites suggest that good quality chest compressions were being performed and with reduced variability. However, the greater variability with ventilation volume for Stage 2 suggests this skill needs frequent practice. This observed significant improvement in infant CPR following the use of feedback has been reported by other authors [22].

The Stage 2 questionnaire results relating to the question; ‘How easy was it to practice on the manikins left with you after Stage 1?’ suggests there was a positive response with an overall mean score of 8.2 (sd = 1.7). The Stage 2 questionnaire results relating to the question; ‘Did the objective feedback help you improve your CPR performance?’ suggests there was a strong positive response with an overall mean value of 8.5 (sd = 1.8). This strong positive result was supported with universal support from the EMS site co-ordinators who suggested that objective feedback motivated participants to practice. The questionnaire results suggest that the objective feedback was most appreciated at Filipstad (mean = 9.0, sd = 2.1) where the manikins appear to have been used the most and least appreciated at Chur (mean = 7.3, sd = 2.9).

### Competition and motivation

All the EMS site co-ordinators stated after Stage 2 that they found it hard to motivate their colleagues to train between Stage 1 and Stage 2. Most wanted to motivate their staff ‘face to face’ but conflicting time pressures, operational obligations and other training requirements meant this was hard to do in practice. The site co-ordinators from the EMS sites covering larger geographic areas also stated that monitoring the training was made considerably harder when the manikins were moved to different areas. The Stage 2 questionnaire results relating to the question; ‘Did the competition between countries motivate you to practice?’ suggests there was a modest response with an overall mean value of 5.8 (sd = 2.9). Filipstad appeared to be the most positive (mean = 8.0, sd = 2.5) and Chur the least (mean = 2.8, sd = 2.2). The site co-ordinators at the end of Stage 2 were also undecided with 4 believing the ‘international’ competition had motivated their teams and 6 that it hadn’t.

The Stage 2 questionnaire results relating to the question; ‘Did the competition between colleagues motivate you to practice?’ suggests there was a more positive response with an overall mean value of 6.0 (sd = 2.7). Again, Filipstad appeared to be the most positive (mean = 8.0, sd = 2.1) and Chur the least (mean = 3.7, sd = 2.4). Paris, Copenhagen and Locarno also had mean scores >6 indicating a positive response. The majority of the site co-ordinators thought that competition between their colleagues increased motivation to practice. The only exception was Amersfoort where the site co-ordinator stated that this aspect was unfortunately not emphasised with the participants during the study.

## Conclusions

This study suggests that the use of real time feedback appears to significantly improve the overall CPR performance of the participants for both the adult and the infant OHCA assessments especially when frequently practiced. The availability of objective feedback when undertaking manikin training to improve CPR performance can have a positive and motivational impact on CPR quality training and could have a positive effect on ‘Educational Efficiency’ as part of the Utstein Formula for Survival.

Questionnaire data from Stage 2 suggests that competition between colleagues at each EMS site was a positive motivator for participants to practice while competition between different sites appears to have had a smaller positive impact on motivation of the participants to practice on the manikins between Stage 1 and Stage 2 of the study. The majority of the EMS site co-ordinators however thought that competition between colleagues at each EMS site had helped motivate their teams to practice. Additional work needs to be done before any firm conclusions can be drawn but the results from this study suggest that competition might have a useful role to encourage motivation and ‘Local Implementation’ of CPR quality training as part of the Utstein Formula for Survival.

## Limitations

Resource constraints and practical considerations meant that a ‘convenience sample’ at each site had to be used and was limited to 10 crews (20 individuals) at each stage of the study. Ideally, a larger sample size would have been preferable.

The questionnaires used for each stage of the study were translated into the local languages. The discussion guide, however, was written in English and language limitations meant that some discussion guide questions were not consistent with the questionnaire questions. This was most evident with questions where a Likert scale was used in the questionnaire but only a binary yes/no question was used in the discussion guide.

## Abbreviations

BVM: Bag Valve Mask
CPR: Cardio Pulmonary Resuscitation
EMS: Emergency Medical Service
ERC: European Resuscitation Council
OHCA: Out of Hospital Cardiac Arrest
PH- SIG: Pre-Hospital Special Interest Group
QCPR: Quality Cardio Pulmonary Resuscitation
